# A Visible Light-Induced and ROS-Dependent Method for the Rapid Formation of a MOF Composite Membrane with Antibacterial Properties

**DOI:** 10.3390/ijms24021520

**Published:** 2023-01-12

**Authors:** Shanshan Zhang, Dongliang Liu

**Affiliations:** College of Chemistry and Chemical Engineering, Donghua University, Shanghai 201620, China

**Keywords:** reactive oxygen species, visible light, dopamine polymerization, mild conditions, metal-organic framework, antibacterial membrane

## Abstract

The diverse application potential of metal-organic framework (MOF) materials are currently limited by their challenging and complicated preparation processes. In this study, we successfully developed a novel strategy for the rapid synthesis of a sustainable MOF composite membrane under neutral conditions with improved physicochemical and antibacterial properties. Our reaction pipeline comprised visible light that induced the production of reactive oxygen species (ROS) from ZIF-8 particles, which facilitated the rapid oxidative polymerization of dopamine to polydopamine. The physicochemical properties of the composite membrane were assessed using imaging methods, including scanning and transmission electron microscopy, X-ray photoelectron spectrometry, and nitrogen adsorption/desorption; its antibacterial effects against *Staphylococcus aureus*, *Escherichia coli*, and *Pseudomonas aeruginosa* were measured using optical densitometry. The bactericidal potency of the synthesized membrane was >99% against all tested strains under the conditions of simulated sunlight. Moreover, the composite membrane retained its structural integrity and antibacterial effect after multiple cycles of use and recovery, showcasing remarkable stability. Overall, this study displays a ROS-mediated method for the rapid preparation of sustainable MOF composite membranes under neutral conditions with optimal physicochemical characteristics, antibacterial properties, and performance. Our study provides insights into the use of membrane materials as design platforms for a range of diverse practical applications.

## 1. Introduction

Metal-organic frameworks (MOFs) are a series of multifunctional hybrid materials consisting of three-dimensional (3D) crystal networks of metal nodes crosslinked by organic ligands [1]. As typical crystal materials with desirable properties, such as a highly specific surface area, adjustable pore size, and diverse physicochemical properties, MOFs have benefited from an extensive amount of research and applications in several real-world contexts [2,3,4]. New MOF composites, which are obtained by the uniform assembly of MOF particles onto the surface of a base material, have opened exciting avenues toward the research and design of MOF membrane materials [5,6,7,8,9,10]. However, the common methods for preparing MOF membranes, including thermal solvent synthesis, liquid phase epitaxial growth, and the Langmuir Blodgett deposition method, among others [11,12,13,14], present several distinct disadvantages, such as the requirement for further surface functional design [15], the agglomeration of MOF particles, or the formation of a defective lattice, all of which can negatively impact the function of MOF crystals [16,17]. Moreover, such methods are intrinsically complex, while the MOF membranes produced by these strategies possess a weak affinity for their substrate and are considerably unstable, as they lose their desired properties relatively fast. Such technical shortcomings warrant effective resolution before MOF membrane materials can successfully transition to larger-scale industrial applications [18,19,20]. Therefore, there remains a pressing challenge regarding the development of a simple yet efficient and universally compatible assembly method for MOF composite membrane materials, which exploits the advantageous properties of MOFs while avoiding or substantially minimizing the associated disadvantages.

Polydopamine (PDA), a highly crosslinked polymer, is formed by the oxidative polymerization of dopamine (DA) [21]. It exhibits strong adhesion properties to various materials and surfaces (including superhydrophobic surfaces), has a controllable deposition thickness (approximatively tens of nanometers), and possesses satisfactory durability and stability [22,23,24]. There is an abundant range of chemical functional groups, such as catechol, primary amine, and secondary amine, on its surface, which allows for the design of composite materials with diverse functions [25,26,27]. As a polymer with unique properties and benefits, PDA has introduced a positive feedback loop between new and alternative strategies for the functional modification of various substances and the impetus for more extensive research in this field [28,29]. Nevertheless, despite the positive characteristics of PDA, the common formation process that generates the PDA coating, i.e., the oxidation-dependent polymerization of DA, requires incubation in an alkaline (pH 8.5) environment for up to 24 h [30], followed by an additional incubation period for 3–48 h for the subsequent PDA surface functionalization construction [31,32,33]. The demanding environment, limited efficiency, and long duration of this synthesis process limit the practical application potential of PDA by a considerable margin, along with the possibility of building more complex interfaces. Several research groups have proposed that the rapid deposition of DA surface coatings can be achieved within hours or even tens of minutes through irradiation by ultraviolet wave bands of different wavelengths [34,35,36]; however, this process involves a lengthy pretreatment as well as the secondary modification of PDA. Therefore, despite the promising features of this strategy, it currently remains an early-stage method of limited efficiency and applicability.

**Figure 1:**
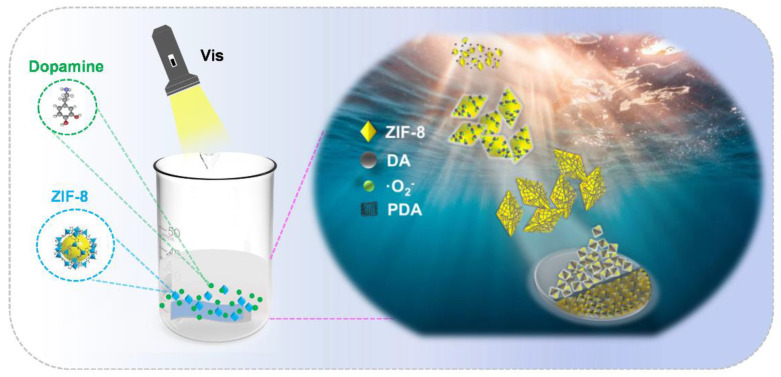
Schematic representation of rapid assembling MOF composite membrane strategy.

Inspired by the caveats of the existing MOF membrane-producing methods, we here propose a strategy whereby the rapid polymerization of DA is incorporated into the preparation process of MOF membrane materials. The oxidation-induced reaction of DA self-polymerization to PDA was initiated under mild conditions (25 °C and pH 7.0), while the strong adhesion properties of the generated PDA accelerated the rapid assembly of the MOF membrane materials in our system. This resulted in the formation of stable MOF composite membranes with enhanced properties within the span of 4 h. As a class of MOF particles with excellent properties, zeolitic imidazolate framework-8 (ZIF-8) has been widely applied in many fields. Thus, we chose stable ZIF-8 particles [37,38,39] and DA [40] as the assembly materials for our membrane preparation system, while we used a homemade polyacrylonitrile (PAN) membrane as the basal membrane (Figure 1). The ZIF-8 particles coexisted with DA in a neutral (pH 7.0) liquid system under visible light (LED, 517 nm, 30 W) and generated reactive oxygen species (ROS) in a sustainable manner. ROS triggered the self-polymerization of DA to PDA under neutral conditions. Simultaneously, the generated PDA could sufficiently adhere to the basal membrane and mediate the ordered arrangement of the ZIF-8 particles on the membrane substrate, which demonstrated the improved synergistic effects between the experimental materials in our reaction system. Due to the advantages of PDA regarding performance, the obtained composite membrane could maintain its structural stability over a prolonged period. Compared to conventional MOF composite membrane preparation, our process enabled the preparation of ZIF-8/PDA/PAN composite membranes in a considerably shorter amount of time (4 h), which possessed a continuous and uniform surface, with more regular dispersion of the MOF particles across the surface area, as well as more stable performance. Finally, we detected that, under simulated sunlight (LED, 250–800 nm, 300 W), the ZIF-8/PDA/PAN composite membranes could rapidly generate large amounts of ROS, which could effectively disrupt the phospholipid layer of bacteria. Thus, the generated ROS conferred physical antibacterial properties to the generated material membrane without the ROS-producing MOF composite membrane being cytotoxic toward L929 cells. The ZIF-8/PDA/PAN composite membrane displayed optimal broad-spectrum bactericidal effects against the tested bacterial strains of Gram-positive *Staphylococcus aureus* (*S. aureus*), Gram-negative *Escherichia coli* (*E. coli*), and *Pseudomonas aeruginosa* (*P. aeruginosa*). In addition to more favorable reaction conditions, our strategy also provides encouraging evidence for further research into the potential antimicrobial properties of novel MOF composite membrane materials.

## 2. Results and Discussion

### 2.1. Characterization of Physical and Chemical Properties of ZIF-8 Particles

**Figure 2:**
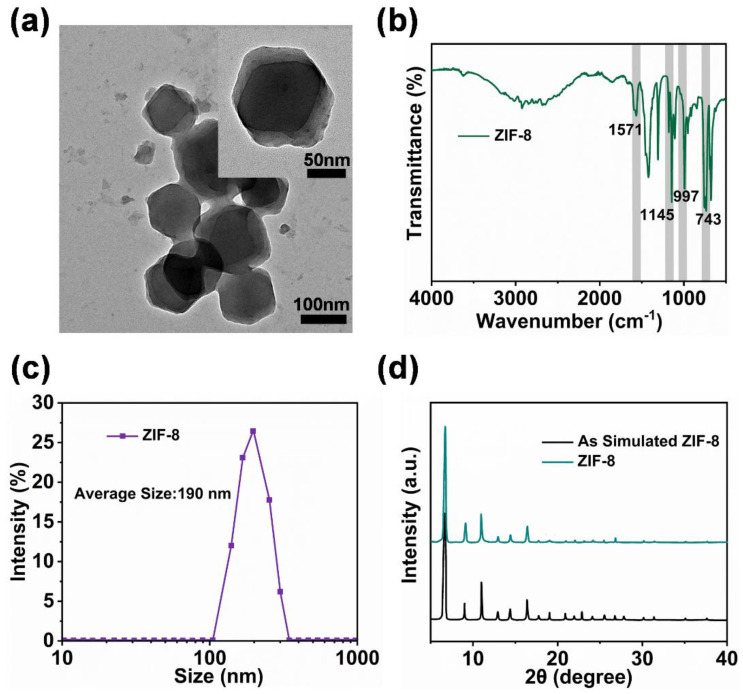
(**a**) TEM images, (**b**) ATR-FTIR spectra, (**c**) DLS measurement image, and (**d**) XRD patterns of ZIF-8 particles.

As displayed in the transmission electron microscopy (TEM) diagram (Figure 2a and illustration), the obtained ZIF-8 particles were of regular polyhedral shape. The particle peaks at 743 cm^−1^, 997 cm^−1^, 1145 cm^−1^, and 1571 cm^−1^, detected by Fourier transform infrared spectroscopy (FTIR) analysis (Figure 2b), were characteristic of the functional group of the ZIF-8 nanoparticles. When combined with the dynamic light scattering (DLS) particle size results (Figure 2c), the average particle size was 190 nm, with a uniform distribution range. Based on the X-ray powder diffraction (XRD) patterns (Figure 2d), the ZIF-8 nanoparticles possessed a clear crystalline structure and high crystallization purity. The above characterization results were consistent with those of a previous study [41] and indicated the successful preparation of the ZIF-8 particles with uniform morphology and stable properties.

### 2.2. Investigation and Analysis of Physical and Chemical Properties of ZIF-8/PDA/PAN Composite Membrane

**Figure 3:**
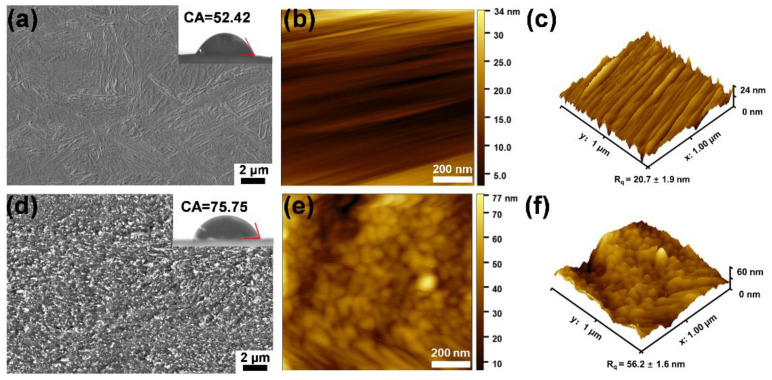
SEM images of basal membrane (**a**) and ZIF-8/PDA/PAN composite membrane (**d**), and the illustrations of their CA, respectively. The 2D and 3D AFM images of basal membrane (**b**,**c**) and ZIF-8/PDA/PAN composite membrane (**e**,**f**).

The ZIF-8/PDA/PAN composite membranes were rapidly assembled under mild conditions (Figure 1). Observations of the membrane surface via scanning electron microscopy (SEM) showed that the basal membrane had a smooth folded surface morphology (Figure 3a), with a root mean square roughness (R_q_) equal to 20.7 ± 1.9 nm (Figure 3b,c). In turn, the surface of the composite membrane was rough, compact, regular, and continuous (Figure 3d). Based on these observations, we could preliminarily determine that the ZIF-8 nanoparticle assembly on the surface of the composite membrane was not characteristic of simple surface adhesion or stacking but more closely resembled codeposition with PDA. When combined with 2D and 3D graphics of atomic force microscopy (AFM; Figure 3e,f), the surface of the ZIF-8/PDA/PAN composite membrane had a grainy bulge, with an R_q_ of 56.2 ± 1.6 nm (Table S1). This value was considerably smaller than the average ZIF-8 particle size (190 nm), which further supported our hypothesis that the ZIF-8 nanoparticles were coassembled concomitantly with PDA polymerization. The roughness of the composite membrane surface increased approximately three times, greatly enhancing the hydrophilicity of the substrate material. Therefore, after codeposition with the ZIF-8 particles and PDA, the membrane surface contact angle (CA) increased from 52.42° to 75.75° (Figure 3a,d). The increase in the surface roughness and CA of the membrane material facilitated the adhesion resistance of the subsequent composite membrane during the initial contact with micropollutants in practical applications.

**Figure 4:**
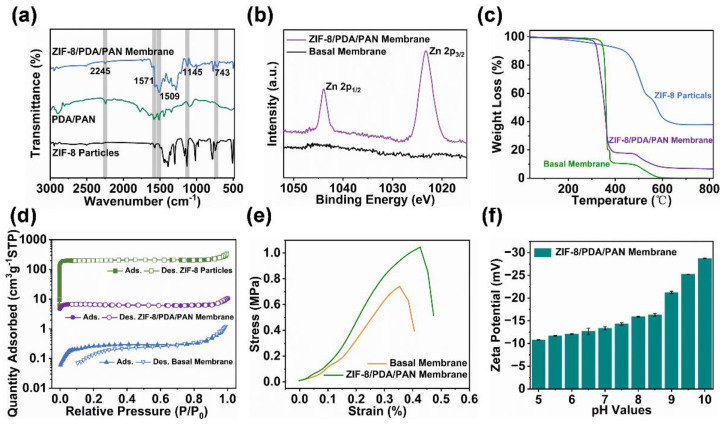
(**a**) ATR-FTIR spectra of the ZIF-8 particles, PDA/PAN membrane, and ZIF-8/PDA/PAN membrane. (**b**) XPS spectra of the zinc element of the basal and ZIF-8/PDA/PAN membranes. (**c**) TG curves and (**d**) N_2_ adsorption-desorption isotherms of the ZIF-8 particles, basal membrane, and ZIF-8/PDA/PAN membrane. (**e**) Stress-strain curves of basal and ZIF-8/PDA/PAN membranes. (**f**) Zeta potential of the ZIF-8/PDA/PAN membrane.

In order to further delineate the deposition state of the ZIF-8 nanoparticles on the membrane surface, we conducted a series of investigations on the properties of the ZIF-8/PDA/PAN composite membranes. The ATR-FTIR spectrum of the MOF composite membrane (Figure 4a) showed multiple strong absorption peaks, among which 2245 cm^−1^ represents the absorption peak of C≡N in the basal membrane (PAN), and the characteristic peak at 1509 cm^−1^ represents the bending vibration of the C−H bond in PDA. The distinct peaks at 1571 cm^−1^, 1145 cm^−1^, and 743 cm^−1^ were attributed to the stretching vibration of the characteristic functional groups in the ZIF-8 particles. These results indicated that the composite membrane was successfully coated with PDA and further loaded with ZIF-8 particles. According to the X-ray photoelectron spectrometry (XPS) analysis of the zinc (Figure 4b), the zinc peaks near the binding energies of 1022 eV and 1045 eV were evident, which confirmed the presence of ZIF-8 particles in the composite membrane. It is worth noting that the two peaks in the element map belonged to narrow peaks; hence, the zinc in the composite membrane was concentrated in the polyhedral coordination structure of the ZIF-8 crystal, which confirmed that the assembly process did not negatively impact the crystal structure of the MOF particles (Figure S1). We next used thermogravimetric (TG) tests to investigate the thermal stability and the ZIF-8 particle-loading of the ZIF-8/PDA/PAN composite membrane. As shown in Figure 4c, the observed reduction in membrane mass at temperatures lower than 300 °C originated from the loss of water from the material, while the sharp decrease in mass between 300 and 400 °C was due to the partial decomposition of the basal membrane. The loss of mass detected between 400 and 600 °C could be attributed to two factors: (a) the degradation of the basal membrane structure, mostly due to the decomposition of the ligands in the ZIF-8 particles, and (b) the mass lost as a result of the collapse of the MOF framework structure. Based on our analysis of the TG results, the final residual masses of the ZIF-8 particles and the ZIF-8/PDA/PAN composite membrane were 38.2 wt.% and 6.7 wt.%, respectively. Based on the data measured through TG, we concluded that the ZIF-8/PDA/PAN composite membrane was rapidly assembled with the assistance of PDA and was loaded with approximately 17.54 wt.% of ZIF-8 particles.

We subsequently investigated the adsorption properties of the basal membrane, the ZIF-8 particles, and the ZIF-8/PDA/PAN composite membrane. The N_2_ adsorption-desorption test results are shown in Figure 4d. The BET surface area of the ZIF-8/PDA/PAN composite membrane was 24.83 m^2^/g, which had increased when compared with the BET surface area of the basal membrane (1.10 m^2^/g). The difference in surface area was attributed to the successful assembly of the ZIF-8 particles on the surface of the MOF membrane (Table S2). The adsorption performance of the composite membrane was also enhanced to some extent. These results further supported our previous conclusion regarding the retention of an intact multistructure of the ZIF-8 particles assembled in the composite membrane, and that the particle assembly mode was dependent on PDA polymerization rather than on surface attachment. Moreover, the introduction of the ZIF-8 particles and PDA also improved the tensile strength and mechanical properties of the MOF composite membrane (Figure 4e). The Zeta potential results highlighted that the surface potential of the ZIF-8/PDA/PAN composite membrane was negative with a trend of gradual increase in the range of pH 5–10 (Figure 4f). Therefore, the ZIF-8/PDA/PAN composite membrane had antipollution potential, and the related performance needed to be analyzed according to the specific application environment.

### 2.3. Mechanism Analysis and Verification of ZIF-8/PDA/PAN Composite Membrane Constructed by ZIF-8 Particle Assembly and DA Codeposition

**Figure 5:**
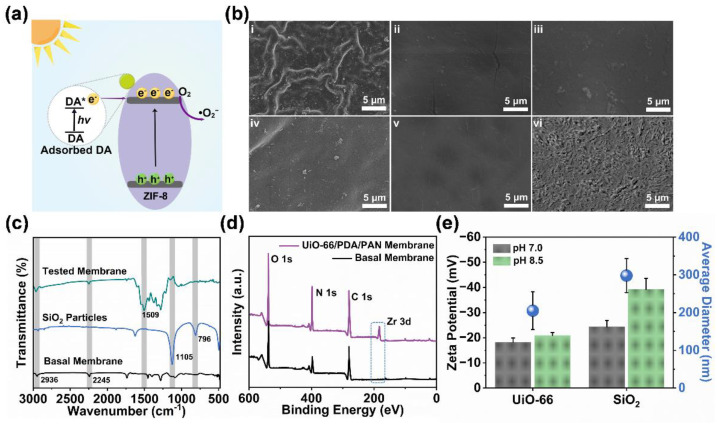
(**a**) Schematics of the co-operative deposition between ZIF-8 and DA; (**b**) SEM images of membranes under (i) UV irradiation, pH 7.0; (ii) dark, pH 7.0; (iii) visible light, pH 8.5; (iv) dark, pH 8.5; (v) visible light, pH 7.0, SiO_2_; and (vi) visible light, pH 7.0, UiO-66; (**c**) zeta potential and average particle size diagram of SiO_2_ and UiO-66 particles; (**d**) ATR-FTIR spectra of the SiO_2_ particles assembled into the new basal membrane; (**e**) XPS spectra of the UiO-66 particles assembled into the new basal membrane.

Our system for the rapid assembly of the ZIF-8/PDA/PAN composite membrane depends on several key variables, such as visible light, DA polymerization, ZIF-8 particles, and the pH of the reaction system. Here, we discuss the synthesis mechanism of the ZIF-8/PDA/PAN composite membrane based on the different combinations of these variables. First, DA, as a polyphenol derivative that is rich in multiple phenol functional groups, can form charge transfer complexes with certain semiconductor materials [42,43] and, thus, expands the specific application of semiconductor materials in the visible light absorption bands. According to the literature [44], ZIF-8 has typical semiconductor material properties. Therefore, we used the imidazole ring of the organic ligand of ZIF-8 and the NH_2_ group on DA to construct a charge transfer complex according to the above theory. The -NH_2_ in the DA was used as the electron donor, and the charge complex formed was the key site for the subsequent π–π electron provider-acceptor transference. Interestingly, the π–π stacking effect occurred between the organic ligand of the ZIF-8 particles and the benzene ring in the DA. As shown in Figure 5a, during the initial stage of the reaction, due to the natural adsorption advantages of the MOF particles, the DA monomer was quickly adsorbed to the surface of ZIF-8. The DA monomer subsequently acted as an electron transfer medium to transfer the photogenerated electrons from the DA molecules to the conduction band of ZIF-8. The photogenerated electrons reduced O_2_ to ROS during the transfer process. This explains why ZIF-8 did not absorb in the visible light bands but produced ROS (Figure S2). In the subsequent reaction, the ROS produced by the system acted as an oxidizing agent for DA, triggering the rapid oxidative polymerization of DA to PDA under pH-neutral conditions. Thereafter, the continuously produced PDA acted as an “adhesive” that enabled the ZIF-8 particles to be fixed at the basal membrane, thus generating the dense continuous ZIF-8/PDA/PAN composite membrane.

We selected different synthesis conditions and materials to explore the described mechanism and improve the preparation conditions of the MOF composite membranes. UV light (λ = 365 nm) sources with the same power as that of visible light could promote the generation of ROS in sufficient amounts (Figure S3). When UV light sources were employed for the preparation of the composite membranes, we found that almost no ZIF-8 particles were deposited on the basal membrane, as shown in Figure 5b-i. This was likely because the excessive ROS caused a surge in the self-polymerization rate of the free DA molecules in the system into PDA, resulting in an inability to participate in the ordered assembly of the ZIF-8 particles on the membrane surface. Moreover, no evident deposition of the MOF particles in the basal membrane was observed under dark conditions (Figure 5b-ii), as ROS, which otherwise mediated the autopolymerization of DA, could not be generated in this system in the dark. Notably, the pH of the reaction system was also essential. Under an alkaline pH of 8.5 (general condition for DA aggregation), the ZIF-8 particles were rarely deposited on the basal membrane, despite the presence of visible light. The reason was that, in this system, the ROS largely promoted the polymerization of DA, which, in turn, reduced the efficiency of the mutual synergy between DA and the ZIF-8 particles (Figure 5b-iii). However, in the absence of any light source, the polymerization process of DA was slow (usually 24 h), even at a pH of 8.5, and, as such, the collaborative deposition of PDA and the ZIF-8 particles could not be completed efficiently and very little MOF particles were deposited on the basal membrane (Figure 5b-iv). Based on these observations, it could be argued that the controlled rate of ROS generation and the role of DA as a “connecting link” are crucial for the successful preparation of MOF composite membranes.

In order to corroborate the functional relevance of ROS in the reaction system, two particle materials with similar properties, SiO_2_ and UiO-66 (both were purchased from Guangdong Huankai Sci&Tech. Co., Ltd., Guangzhou, China) were selected (Figure 5c). As a common and stable MOF material, UiO-66 can also generate ROS in a similar manner to ZIF-8 under visible light [45]. However, SiO_2_ cannot produce ROS. Therefore, when these particles were used to prepare composite membranes within our reaction system, we correspondingly observed no deposition of SiO_2_ particles in the composite membranes (Figure 5b-v), while the UiO-66 particles generated a rough surface similar to the ZIF-8/PDA/PAN composite membrane (Figure 5b-vi). Supporting this finding, the ATR-FTIR did not return the characteristic functional group absorption peak of SiO_2_ in the membrane materials (Figure 5d), in contrast to the XPS element analysis results, which displayed the characteristic peak of UiO-66 (Figure 5e). This indicates that ROS generation is an essential step for successful MOF particle deposition on the surface of the composite membrane. Moreover, the composite membrane prepared in the UiO-66 system displayed a synergistic effect with the polymerization of DA, which was successfully assembled on the surface of the composite membrane. Simultaneously, these findings also further supported the wider application potential of our method for synthesizing composite MOF membrane materials. Overall, these results provide encouraging initial evidence for the feasibility of our strategy of utilizing the synergistic codeposition of polymerized DA and ZIF-8 assembly under visible light-induced conditions for the preparation of MOF composite membranes.

### 2.4. Antibacterial Mechanism and Application of ZIF-8/PDA/PAN Composite Membrane

**Figure 6:**
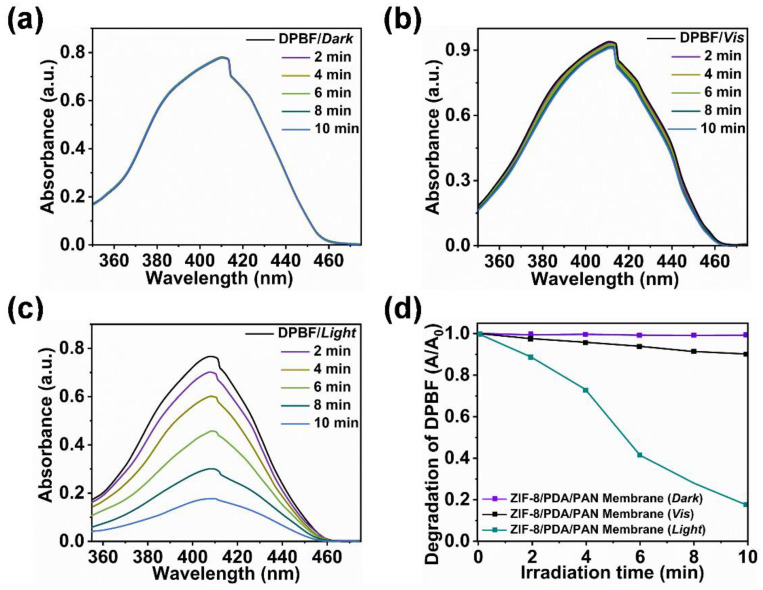
UV-vis spectrum of the ZIF-8/PDA/PAN composite membrane in DPBF solution under (**a**) dark, (**b**) visible light, and (**c**) simulated sunlight conditions for 2 min, 4 min, 6 min, 8 min, and 10 min, respectively; (**d**) normalized degradation curve of the DPBF solution absorbance change at 410 nm under different light conditions.

In order to characterize the antibacterial performance of the ZIF-8/PDA/PAN composite membrane in detail, we first used 1,3-Diphenylisobenzofuran (DPBF) as a chemical probe to quantitatively analyze the ability of the composite membranes to produce ROS under different conditions. As shown in Figure 6a,b, the absorbance of the DPBF solution did not change considerably between the conditions of no light and visible light without irradiation, which indicated that the composite membrane produced no, or only a small amount of, ROS under these two conditions. However, under the conditions of simulated sunlight irradiation (Figure 6c), the absorbance of the DPBF solution decreased significantly within 10 min of exposure. By examining the normalized degradation curve of the DPBF solution (Figure 6d), we could deduce that the composite membrane could only produce a limited ROS amount when exposed to visible light, while this ability was greatly enhanced by exposure to simulated sunlight. Together, these data suggest that simulated sunlight exposure substantially improves the ROS yield from the composite membrane. 

**Figure 7:**
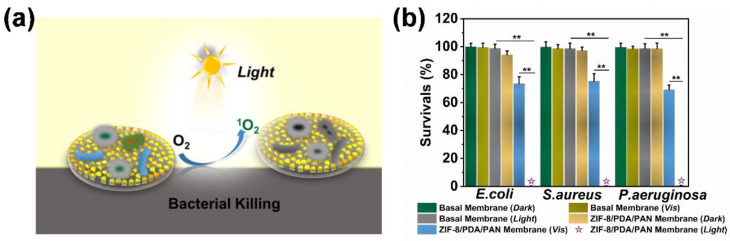
(**a**) Schematic diagram of the photodynamic bactericidal mechanism of the ZIF-8/PDA/PAN composite membrane; (**b**) sterilization efficiency of ZIF-8/PDA/PAN composite membrane on *E. coli*, *S. aureus*, and *P. aeruginosa* (*n* = 3, ** *p* < 0.01).

Here, the photogenerated ROS are an effective broad-range bactericidal, as they can irreparably disrupt the phospholipid bilayer of bacteria and consequently kill such pathogenic microbes [46] without raising concerns over the emergence of bacterial resistance, owing to the nature of its mechanism of action [47]. Thus, the composite membrane could also act as a photodynamic antibacterial platform (Figure 7a). We employed the ZIF-8/PDA/PAN composite membrane within in vitro experiments whereby the material was coincubated with *E. coli*, *S. aureus*, or *P. aeruginosa* cultures (Figure 7b and Figure S4). Our findings suggested that the ZIF-8/PDA/PAN composite membranes could kill >99% of the bacterial population for all three of the tested strains under the conditions of simulated sunlight for two hours, showcasing the excellent broad-spectrum antibacterial performance of the composite membranes.

**Figure 8:**
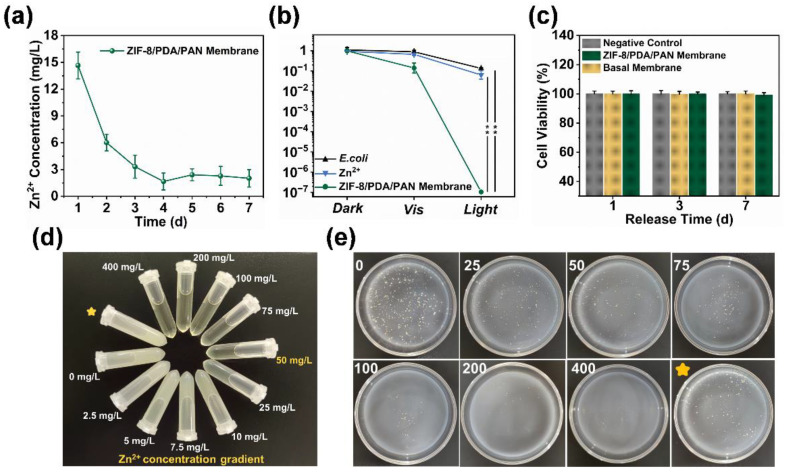
(**a**) Release concentrations of Zn^2+^ in the ZIF-8/PDA/PAN composite membrane over 7 days; (**b**) under different conditions, the killing effects of Zn^2+^ and the ZIF-8/PDA/PAN composite membrane on *E. coli* (*n* = 3, ** *p* < 0.01); (**c**) the effects of different Zn^2+^ concentrations on L929 cell activity; (**d**) camera pictures of Zn^2+^ with different concentrations; (**e**) photographs for the killing effects of different Zn^2+^ concentrations on *E. coli* (the yellow star was the experimental extract concentration; the Zn^2+^ minimum inhibitory concentration labeled yellow).

In order to exclude the potential influence of the bactericidal effect of Zn^2+^ [48], we monitored the release of Zn^2+^ from the ZIF-8/PDA/PAN composite membranes over seven days. As the leaching trend of Zn^2+^ shows (Figure 8a), the composite membrane rapidly released Zn^2+^ over the first two days, which then gradually decreased to a stable trend. The released Zn^2+^ reached a peak value of 14.653 mg/L on day 1, which most likely originated from a small amount of hydrolyzed ZIF-8 precursor adsorbed on the surface of the membrane [49]. The release of Zn^2+^ gradually plateaued around day 4 and stabilized at approximately 2.024 mg/L by day 7. We compared the in vitro antibacterial performance of the ZIF-8/PDA/PAN composite membrane with that of the membrane releasing Zn^2+^ at equivalent concentrations (Figure 8b). The results showed that free Zn^2+^ in the solution exhibited weak bactericidal activity against *E. coli*. Of note, the cytotoxicity tests conducted on the extracts of the ZIF-8/PDA/PAN composite membranes at different time periods demonstrated no adverse cytotoxic effect of Zn^2+^ on the cocultured L929 cells over the course of seven days (Figure 8c). The released Zn^2+^ also did not affect the subsequent growth and proliferation of the cells (Figure S5). As shown in Figure 8d, the actual leakage of Zn^2+^ was markedly lower than both the minimum Zn^2+^ inhibitory (50.0 mg/L) and minimum bactericidal (250 mg/L) concentration of the metal ion (Table S3). Based on the experimental results shown in Figure 8e, the concentration of Zn^2+^ leached from the composite membrane did not have an obvious bactericidal effect. Taken together, we highlight that the bactericidal properties of the ZIF-8/PDA/PAN composite membrane in our system were primarily conveyed by the photodynamic antibacterial effect of the ROS (Figure 7a), showcasing the good biocompatibility and remarkable bactericidal properties of the composite membrane. 

### 2.5. Characterization of ZIF-8/PDA/PAN Composite Membrane Stability

**Figure 9:**
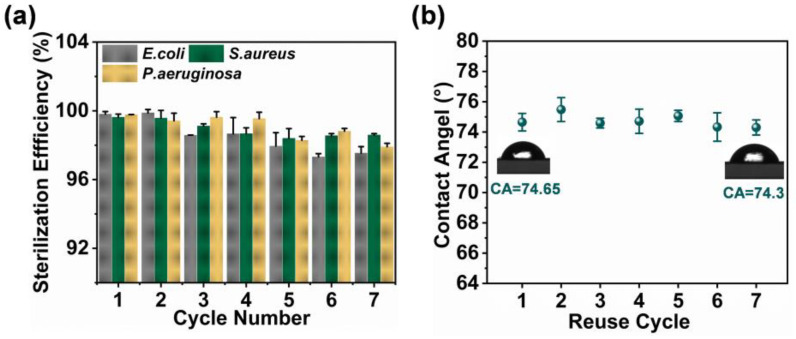
(**a**) Killing efficiencies of the ZIF-8/PDA/PAN composite membrane on *E. coli*, *S. aureus*, and *P. aeruginosa* in 7 cycles; (**b**) changes in the surface CA of the ZIF-8/PDA/PAN composite membrane after 7 cycles of use.

In order to assess the stability of the ZIF-8/PDA/PAN composite membrane after repeated application cycles, we recovered and reused the same prepared composite membrane. The photodynamic antibacterial effect of the ZIF-8/PDA/PAN composite membrane against multiple strains remained at a sufficiently high level (>97%) after seven reuse cycles (Figure 9a). Moreover, according to the CA results of the composite membrane after use, the hydrophilicity of the composite membrane, following repeated applications, slightly differed (Figure 9b). Nevertheless, a CA of 74.30° was still sufficient for the membrane to resist the adhesion of bacteria and some organic foulants. Overall, our findings suggested that the composite membrane’s structural integrity and antibacterial performance remained largely uncompromised between cycles of repeated application and recovery. 

## 3. Materials and Methods

### 3.1. Experimental Materials

PAN (MW = 150,000 Da) was purchased from Jinan Daigang Biological Co., Ltd. (Shandong, China). Ethanol (99.8%), DA (≥98%), zinc nitrate hexahydrate (Zn [NO_3_]_2_⋅6H_2_O, 99%), and N-N dimethylformamide (DMF, >98%) were purchased from Sinopharm Reagent Co., Ltd. (Shanghai, China). The 2-methylimidazole (2-Hmim, 98%), acetonitrile (99%), and DPBF (≥97%) were purchased from Aladdin Reagent Co., Ltd. (Shanghai, China). Nutrient Agar and DMEM were purchased from McLean Biotechnology Co., Ltd. (Beijing, China). The Gram-negative *E. coli* (ATCC 8739) and *P. aeruginosa* (ATCC 9027), and the Gram-positive *S. aureus* (ATCC 6538) were purchased from Guangdong Food Safety Culture Preservation Center (Guangdong, China); the L929 cells were purchased from the Cell Bank of the Chinese Academy of Sciences (Shanghai, China). PAN-basal membranes and deionized water were made in the laboratory. All reagents were used as provided, without further modifications.

### 3.2. Preparation of ZIF-8/PDA/PAN Composite Membrane

The preparation of the ZIF-8 particles was carried out as previously described [50]. Briefly, 0.372 g of Zn [NO_3_]_2_⋅6H_2_O and 0.205 g of 2-Hmim were each dissolved in 50 mL methanol solution in a separate container. The two solutions were mixed immediately after resuspension into a beaker under continuous stirring until an emulsion appeared. Following particle crystallization, the produced ZIF-8 particles were collected, centrifuged, cleaned, and vacuum-dried at 25 °C for further use. To codeposit the ZIF-8 particles and PDA on the PAN-basal membrane, prior to all experiments, the basal membrane (2 cm × 2 cm; rinsed with DI water in advance) was firmly fixed at the bottom of a beaker. Thereafter, the prepared ZIF-8 particles (2 mg/mL) were applied on top of the basal membrane together with DA (2 mg/mL) and 20 mL of Tris-HCl solution (10 mM, pH 7.0 or 8.5). The mixture was subsequently placed into a shaker and incubated for 4 h (37 °C, 110 r/min) under visible light irradiation conditions. The synthesized sample was soaked in ethanol solution (99%) and underwent ultrasonic treatment (50 kHz), after which it was rinsed with DI water for 30 min to remove residual substances from the surface. The produced ZIF-8/PDA/PAN composite membrane was vacuum-dried overnight. All synthesized membranes were stored in the dark at 4 °C until use.

### 3.3. Characterization of ZIF-8 Nanoparticles and ZIF-8/PDA/PAN Composite Membrane Physicochemical Properties

SEM (Sigma 300, Zeiss, Oberkohen, Germany), TEM (SU8020, Hitachi, Tokyo, Japan), and DLS (Zetasizer Nano ZS90, Malvern, UK) tests were employed to observe the surface morphology and particle size distribution of the analyzed samples. Material crystallization, characteristic functional groups, and element distribution were examined by XRD (SmartLab, Rigaku, Tokyo, Japan), ATR-FTIR (8400S, SHIMADZU, Kyoto, Japan), and XPS (ESCALAB 250Xi, Thermo Scientific, MA, USA), respectively. Surface roughness of the composite membrane was assessed by AFM (Dimension Fastscan, Bruker, Billerica, USA), while nitrogen adsorption/desorption (Autosorb-IQ, Quantachrome, FL, USA) was used to detect the pore structure and specific surface area of the samples. TG (Netzch Sta 449c, Jupiter, Selbu, Germany) analyses were employed for calculating the specific mass content of the different components. CA (QSPJ-360, Jinshengxin Co., Ltd., Shenzhen, China) and tensile property tests were used to evaluate the hydrophilicity and mechanical properties of the material, respectively. The change in surface potential of the composite membrane in different pH environments was monitored by Zeta potential (Zetasizer Nano ZS90, Malvern, UK) tracking.

### 3.4. ROS Yield Analysis of ZIF-8/PDA/PAN Composite Membrane

DPBF was selected as the chemical probe for detecting ROS in this study [51]. Specifically, 40 μL of DPBF (4 mM) was absorbed and added into 3 mL of acetonitrile solution. The ZIF-8/PDA/PAN composite membrane was then fixed at the bottom, and visible light was provided according to experimental requirements by a sunlight-simulating source for irradiation (2, 4, 6, 8, and 10 min). The initial absorbance of the DPBF solution at 410 nm, recorded by an ultraviolet-visible spectrophotometer, was set as A0. The absorbance at 410 nm under different irradiation times was recorded as An (n = irradiation duration). The ultraviolet and no-light groups were given and followed the same experimental steps. To test the ROS yield of the system, the solution was placed in an ultraviolet lamp (30 W) irradiation environment at 365 nm for 10, 20, 30, 40, or 50 sections, and its absorbance was subsequently measured by an ultraviolet spectrophotometer. 

### 3.5. Zn^2+^ Release Test of the ZIF-8/PDA/PAN Composite Membrane

The Zn^2+^ release from the ZIF-8/PDA/PAN composite membrane was detected by spectrophotometry, as previously described [52]. Briefly, the prepared composite membrane was immersed in 5 mL PBS buffer (pH 7.2~7.4, Solarbio, Beijing, China) and then oscillated in a shaker incubator for 1–7 d. A sample volume was taken from the container daily, and zinc release (OD_620 nm_) was quantified with zinc reagent spectrophotometry by a microplate reader (Infinite 200PRO, Tecan, Männedorf, Switzerland). The solution in the container was supplemented by a volume of PBS buffer equal to the sampling amount taken to ensure a constant measurement volume. Finally, the specific release amounts of Zn^2+^ were calculated based on the constructed standard curve. 

### 3.6. Bacterial Culture

The selected model strains were *S. aureus*, *E. coli*, and *P. aeruginosa*. First, different strains were inoculated into agar medium and cultured in a 37 °C shaker incubator for 16 h. The bacteria were then collected by centrifugation (5 min at 5000 r/min, 25 °C) and washed thrice with PBS buffer. The collected bacteria were resuspended in PBS buffer, and the bacterial optical density (OD_600 nm_) was measured in a microplate reader. The required concentration of bacteria was subsequently adjusted according to the OD_600 nm_ value measured. The bacterial solution was 10^6^ CFU/mL.

### 3.7. Assessment of Antibacterial Properties of the ZIF-8/PDA/PAN Composite Membrane under Simulated Sunlight

All experiment materials and reagents were sterilized before use. First, the ZIF-8/PDA/PAN composite membrane (2 × 2 cm) was fixed at the bottom of a six-well plate, and the bacterial solution (*S. aureus*, *E. coli*, or *P. aeruginosa*) was subsequently applied on top of the membrane surface at a concentration of 10^6^ CFU/mL (3 mL per well). Plates were incubated at 37 °C for 4 h, after which the experimental sample was exposed to simulated sunlight for 120 min. The light source was maintained at a distance of 0.4 m above the sample. Following illumination, PBS buffer was added to the plate wells to collect the bacterial solution. After washing thrice and diluting the bacteria with PBS buffer, 200 μL of bacterial solution was placed on a Petri dish (diameter: 75 nm) and incubated at 37 °C for 16 h. The OD_600 nm_ value was determined using a microplate reader, and the number of bacteria was adjusted by the optical density. The experimental steps for the visible light and the dark groups were the same as above. 

### 3.8. Cytotoxicity Test

Cell cytotoxicity was measured using a Cell Counting Kit-8 (CCK-8, Solarbio, Beijing, China) test; the L929 cell line was selected for observations in mammalian cells in vitro. Cells were cultured with 10% fetal bovine serum medium (DMEM, Gibco, Billings, MT, USA) at 37 °C and 5% CO_2_ for 3 d. After reaching confluency, the cells were treated with trypsin-EDTA (0.25%) and counted to determine the initial cell number per millimeter. Cells were diluted with DMEM at a working concentration of 2 × 10^5^ cells/mL and inoculated into 96-well plates (100 μL per well) for incubation in the presence of extract containing different ZIF-8/PDA/PAN composite membranes. Cells cultured in DMEM-only medium were used as blank controls. 

### 3.9. Statistical Analysis

All data were recorded by means of three replicates, with standard deviation. The results were analyzed for variance using the GraphPad Prism5.0 statistical analysis software and visualized with Originlab 2022.

## 4. Conclusions

This research provides initial evidence in support of a sustainable method for the rapid formation of a multifunctional MOF membrane based on the assembly of DA—assisted MOF particles under mild conditions (25 °C and pH 7.0). The MOF composite membrane prepared according to our current protocol exhibited favorable physical and chemical properties while also demonstrating potent photodynamic bactericidal performance. The structural integrity and bactericidal properties of the synthetic membrane were retained after multiple cycles of recovery and reuse. Our proposed strategy enables the design and application of sustainable and broad-spectrum antimicrobial MOF membrane materials. Moreover, the MOF composite membrane based on PDA and the MOF particle assembly layer allows for a range of functionally diverse designs with promising application potential in future feasibility studies. Finally, the method discussed herein may serve as a reference strategy for the design and preparation of stable and multifunctional MOF membrane materials.

## Data Availability

Data are contained within the article or Supplementary Files.

## Supplementary Materials

The following supporting information can be downloaded at: https://www.mdpi.com/article/10.3390/ijms24021520/s1. Reference [53] are cited in the Supplementary Material.
