# Supplementary material for: A Visible Light-Induced and ROS-Dependent Method for the Rapid Formation of a MOF Composite Membrane with Antibacterial Properties
Source: Int J Mol Sci. 2023 Jan 12;24(2):1520. doi: 10.3390/ijms24021520 (PMC9861057; doi:10.3390/ijms24021520)
Supplement: Supplementary file 1 [file ijms-24-01520-s001.zip › ijms-2134527-supplementary.pdf]

Supplementary Information

A Visible Light-Induced and ROS-Dependent Method for the Rapid Formation of a MOF Composite Membrane with Anti-bacterial Properties

Shanshan Zhang and Dongliang Liu *

College of Chemistry and Chemical Engineering, Donghua University, Shanghai 201620, China

* Correspondence: dlliu@dhu.edu.cn; Tel.: +86-13761896520

Supplementary Table S1. Roughness of experimental samples

Sample	Rq/nm			Average Value / nm	Standard Deviation / nm
Basal Membrane	22.8	18.9	20.4	20.7	1.9
ZIF-8/PDA/PAN Membrane	53.5	57.1	58.0	56.2	1.6

Supplementary Table S2. BET value of experimental samples

Sample	BET value (m^2/g)
Basal Membrane	1.10
ZIF-8	897
ZIF-8/PDA/PAN Membrane	24.83

Supplementary Table S3. The values of MIC and MBC of Zn^{2+} against *E. coli* strain [1].

	MIC (mg/L)	MBC (mg/L)
	E. coli	
Zn^{2+}	50.0	250

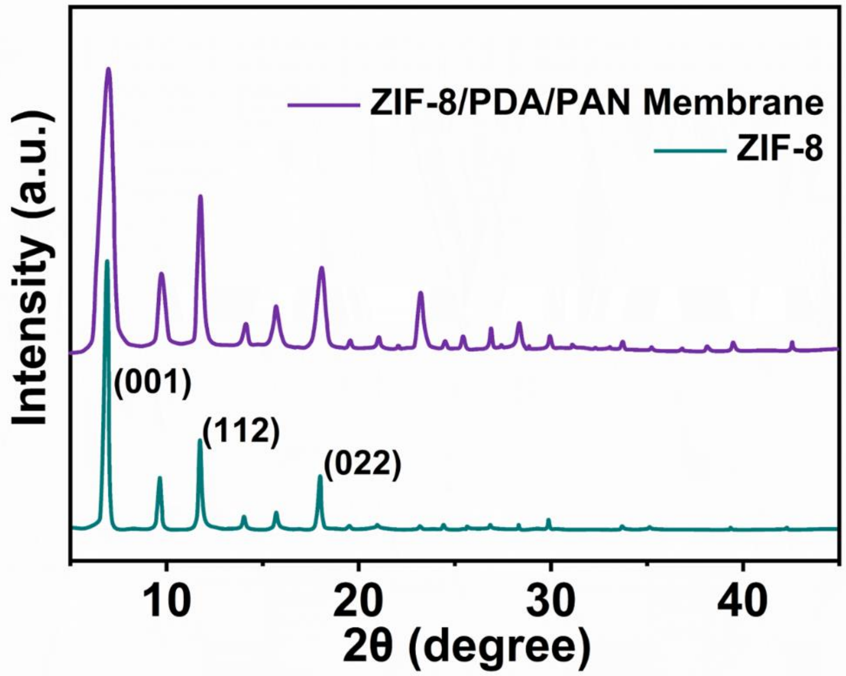

Supplementary Figure S1. *XRD patterns of ZIF–8 Particles and ZIF–8/PDA/PAN composite membrane.*

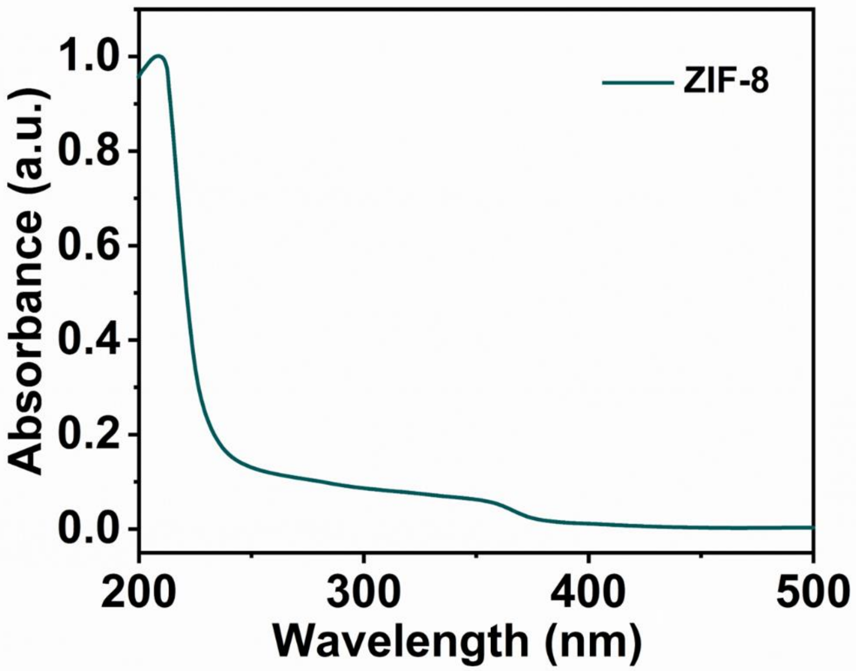

Supplementary Figure S2. *UV–vis diffuse reflectance spectra of ZIF–8* [2].

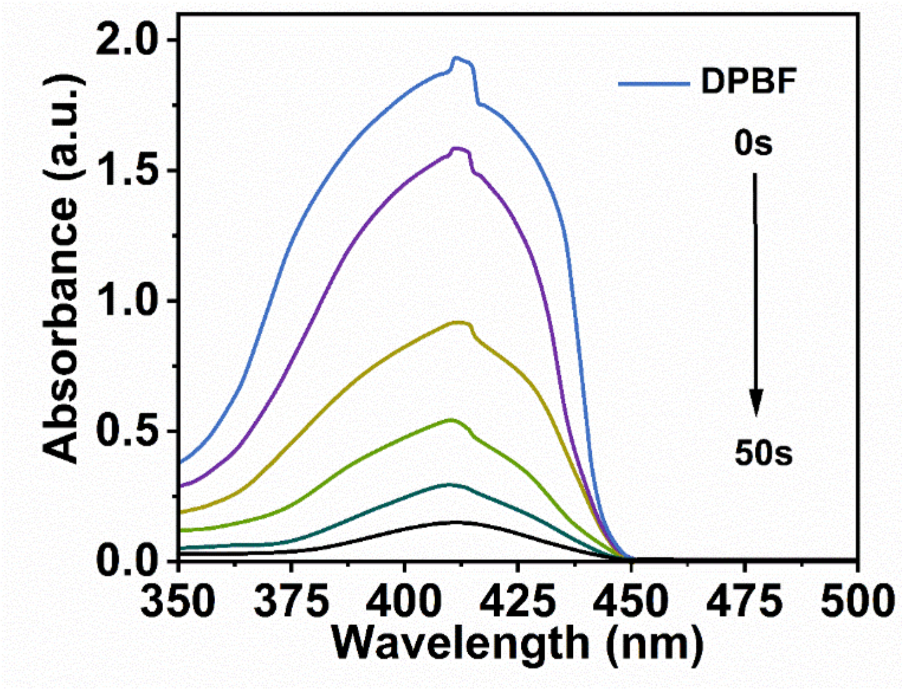

Supplementary Figure S3. *Time–dependent UV–vis spectrum of DPBF, in the presence of ZIF–8, under the illumination of the UV (LED, 365 nm, 30 W) after irradiation at 0s, 10s, 20s, 30, 40s, and 50s.*

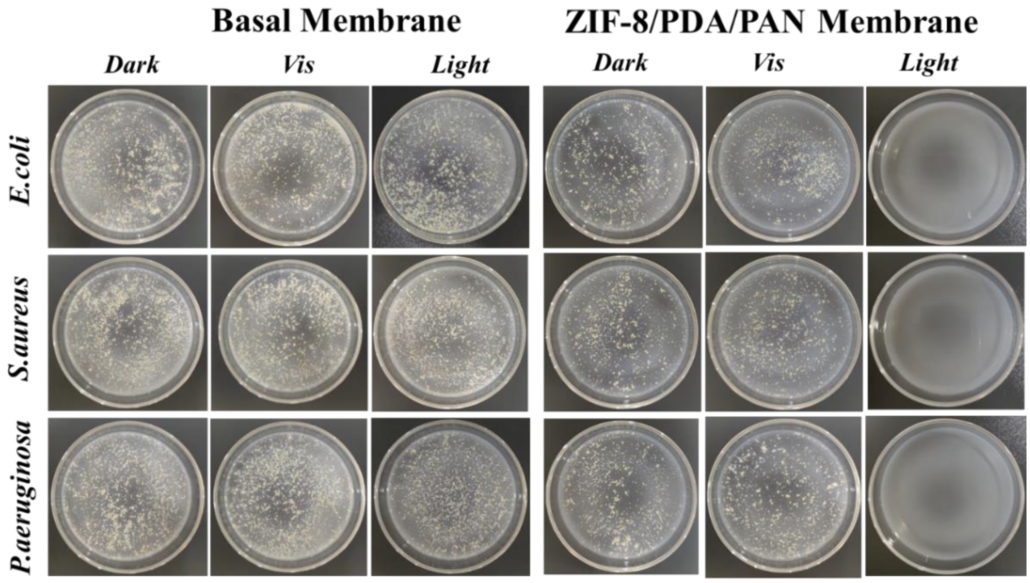

Supplementary Figure S4*. Photos dynamic antibacterial effects of ZIF–8/PDA/PAN composite membrane / basal membrane on different strains* [3].

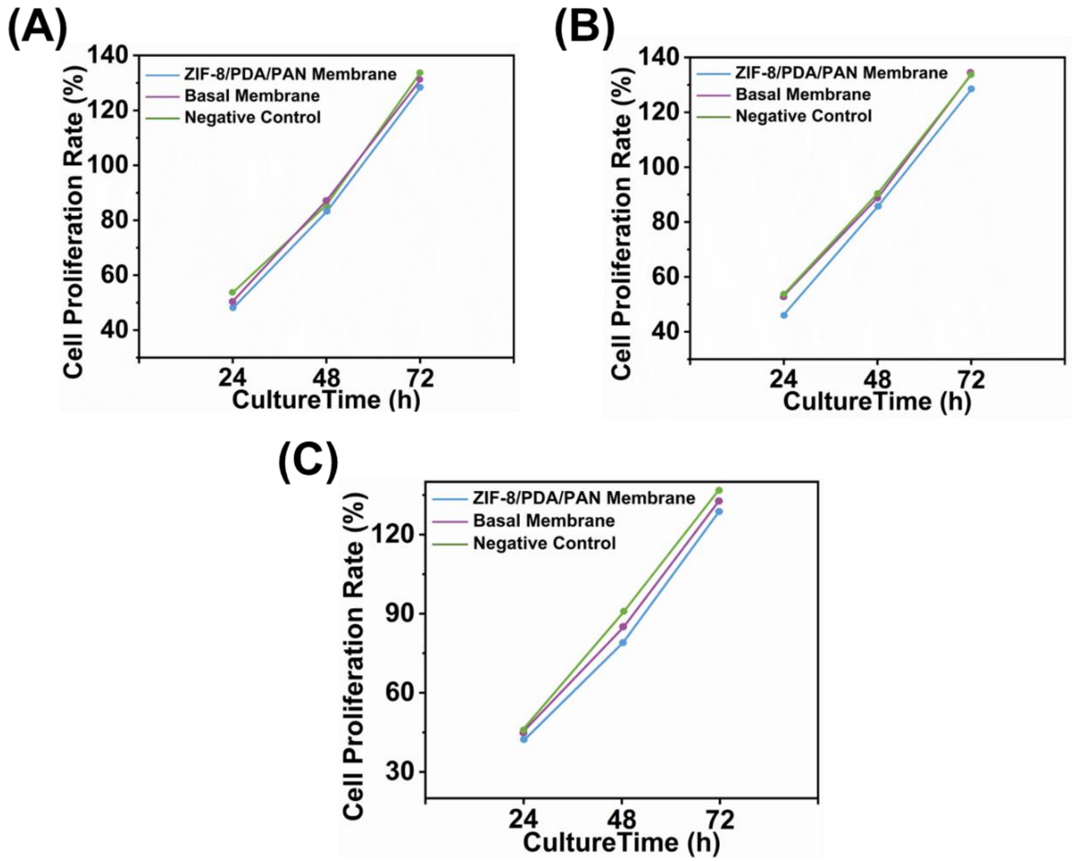

Supplementary Figure S5. *Effects of the extracts of the ZIF–8/PDA/PAN composite membrane at (**A**) 1 day, (**B**) 3 days, (**C**) 7 days on the continuous culture and proliferation of L929 cells.*

Reference

1. Wang, Z.Y.; Qi, J.Y.; Lu, X.H.; Jiang, H.C.; Wang, P.P.; He, M.R.; Ma, J. Epitaxially grown MOF membranes with photocatalytic bactericidal activity for biofouling mitigation in desalination. *J. Membr. Sci*. **2021**, *630*, 119327, doi: 10.1016/j.memsci.2021.119327.
2. Li, P.; Li, J.Z.; Feng, X.; Li, J.; Hao, Y.C.; Zhang, J.W.; Wang, H.; Yin, A.X.; Zhou, J.W.; Ma, X.J.; et al. Metal-organic frameworks with photocatalytic bactericidal activity for integrated air cleaning. *Nat. Commun.* **2019**, *10*, 2177, doi: 10.1038/s41467-019-10218-9.
3. Ding, X.; Duan, S.; Ding, X.; Liu, R.; Xu, F. J. Versatile Antibacterial Materials: An Emerging Arsenal for Combatting Bacterial Pathogens. *Adv. Funct. Mater.* **2018**, *28*, 1802140-1802159, doi: 10.1002/adfm.201802140.
